# Genes significantly associated with lineage II food isolates of *Listeria monocytogenes*

**DOI:** 10.1186/s12864-018-5074-2

**Published:** 2018-09-25

**Authors:** Cary Pirone-Davies, Yi Chen, Arthur Pightling, Gina Ryan, Yu Wang, Kuan Yao, Maria Hoffmann, Marc W. Allard

**Affiliations:** 1 Division of Microbiology, Office of Regulatory Science, Center for Food Safety and Applied Nutrition, U.S. Food and Drug Administration, College Park, MD USA; 2 Office of Analytics and Outreach, Center for Food Safety and Applied Nutrition, U.S. Food and Drug Administration, College Park, MD USA

**Keywords:** Listeria, Comparative genomics, Virulence, Adaptation, Plasmid

## Abstract

**Background:**

*Listeria monocytogenes* is a widespread foodborne pathogen that can cause listeriosis, a potentially fatal infection. *L. monocytogenes* is subdivided into four phylogenetic lineages, with the highest incidence of listeriosis occurring within lineage I followed by lineage II. Strains of *L. monocytogenes* differ in their phenotypic characteristics, including virulence. However, the genetic bases for these observed differences are not well understood, and current efforts to monitor *L. monocytogenes* in food consider all strains to be equally virulent. We use a comparative genomics approach to identify genes and single nucleotide polymorphisms (SNPs) in 174 clinical and food isolates of *L. monocytogenes* that potentially contribute to virulence or the capacity to adapt to food environments.

**Results:**

No SNPs are significantly associated with food or clinical isolates. No genes are significantly associated with food or clinical isolates from lineage I, but eight genes consisting of multiple homologues are associated with lineage II food isolates. These include three genes which encode hypothetical proteins, the cadmium resistance genes *cadA* and *cadC*, the multi-drug resistance gene *ebrB*, a quaternary ammonium compound resistance gene *qac*, and a regulatory gene. All eight genes are plasmid-borne, and most closed *L. monocytogenes* plasmids carry at least five of the genes (24/27). In addition, plasmids are more frequently associated with lineage II food isolates than with lineage II clinical isolates.

**Conclusions:**

We identify eight genes that are significantly associated with food isolates in lineage II. Interestingly, the eight genes are virtually absent in lineage II outbreak isolates, are composed of homologues which show a nonrandom distribution among lineage I serotypes, and the sequences are highly conserved across 27 closed *Listeria* plasmids. The functions of these genes should be explored further and will contribute to our understanding of how *L. monocytogenes* adapts to the host and food environments. Moreover, these genes may also be useful as markers for risk assessment models of either pathogenicity or the ability to proliferate in food and the food processing environment.

**Electronic supplementary material:**

The online version of this article (10.1186/s12864-018-5074-2) contains supplementary material, which is available to authorized users.

## Background

*Listeria monocytogenes* is a facultative intracellular pathogen that causes listeriosis, a predominantly foodborne disease with high case fatality and hospitalization rates (up to 19 and 94%, respectively) [1]. Immunocompromised individuals including the elderly, infants, and pregnant women, are at particularly high risk for contracting invasive listeriosis [2, 3].

Rates of *L. monocytogenes* infection have been difficult to control, in part, due to the widespread dissemination of *L. monocytogenes* in the natural environment. Outside of its animal hosts, *L. monocytogenes* survives as a saprotroph, colonizing diverse natural and urban settings. *L. monocytogenes* is also highly resilient to various stresses. It can survive refrigeration temperatures of 4 °C [4], salt concentrations as high as 13.9% and pH values as low as 4.1 [5, 6]. This capacity for tolerating extreme conditions which inhibit the growth of most foodborne pathogens makes it of particular concern for the food industry. Currently, the U.S. enforces a zero tolerance policy for *L. monocytogenes* in ready to eat foods, such that if a detectable level of *L. monocytogenes* is present in a food (1 CFU *L. monocytogenes* per 25 g food), that food is deemed a hazard to health [7].

*L. monocytogenes* consists of four phylogenetic lineages [8–12] that vary in their ecological, evolutionary, and phenotypic characteristics, including virulence [11–15]. There is strong evidence that strains belonging to lineage I are on average more virulent than those from lineage II. Lineage I strains are more frequently associated with human clinical cases and are predominantly linked to outbreaks involving invasive disease [16]. Additionally, in vitro and animal studies indicate invasion and disease phenotypes are more frequent among lineage I strains compared to lineage II strains [17]. Furthermore, strains from lineage I are highly clonal [18], suggesting that genetic traits important for fitness within the host are under strong selection. Lineage II strains show higher rates of recombination than those from lineage I, which may contribute to an enhanced capacity to adapt to various ecological niches [18]. This increased genomic plasticity among lineage II strains supports the findings from several studies that show lineage II isolates are more frequently isolated from a broad diversity of sources, including foods, than lineage I strains [19, 20]. Strains from lineages III and IV are rarely associated with human disease and are predominantly isolated from animal sources [21, 22].

*L. monocytogenes* is further categorized into 13 serotypes [23] and numerous clonal groups [24, 25] that exhibit heterogeneity in virulence both within and among lineages [14, 24, 25]. Lineage I serotypes 1/2b, 4b, and lineage II serotype 1/2a are responsible for > 95% of human illness [26], while clonal complexes CC1 and CC6 are associated with the highest incidences of infection [14, 27].

Stress tolerance promotes survival in either the host or certain environmental niches and may indirectly influence virulence by increasing isolate abundance in the food supply and in turn, increase the chances of an isolate reaching and infecting a host. Some lineage II strains show an enhanced capacity to survive and grow under various stressors when compared to lineage I strains [13], and serotype 4b isolates show enhanced survival after heat treatment following cold storage when compared to 1/2a isolates [28].

The genetic bases for differences in virulence and stress tolerance are not well understood, although many genes involved in these traits have been documented (for reviews on virulence genes, see [29–32], stress tolerance genes [33–35]). Many strains carrying a truncated internalin A (inlA) show reduced virulence [36, 37], but few genomic markers can reliably predict the capacity for virulence. Most recognized virulence genes (e.g., *inlB, prfA,* and *sigB*) are largely conserved across the species [14, 22, 38], except the pathogenicity island LIPI-3, which is largely restricted to lineage I isolates [38], and the *Listeria* pathogenicity island 4 (LIPI-4), implicated in neural and placental infections [14], is present only in a subset of isolates [14, 38]. Gene losses and truncations among several virulence genes have been observed and may account, in part, for attenuated virulence [14, 22, 38]. However, deletions and truncations are also observed in highly virulent strains, indicating that these mutations are not solely responsible for observed differences in virulence [14, 22, 38]. Comparative genomics studies in *L. monocytogenes* have focused on the association between virulence and clonal groups or sublineages. No large-scale study has examined the association between virulence or stress tolerance genes and isolation source, although data from cell culture and animal model studies indicate that clinical isolates are, on average, more virulent than food isolates [39–41]. Neither has there been a large-scale comparative study that has examined the association between single nucleotide polymorphisms (SNPs) and isolation source. SNPs resulting in a non-synonomous substitution may affect gene function and contribute to observed phenotypic differences among strains.

Thus, to assess genetic differences across the species, we compare the genomes of 174 *L. monocytogenes* isolates to identify genes and SNPs that are non-randomly associated with either food or clinical isolates. Current risk assessment models of *L. monocytogenes* which predict the virulence of strains or the risk of certain foods would benefit greatly from additional genomic markers of virulence or enhanced survival in food [42]. These data will also enhance our understanding of how *L. monocytogenes* adapts to the host and food environments. This information is critical for reducing *L. monocytogenes* in the food supply and treating patients once they are infected.

## Methods

### Isolate selection

We selected 169 clinical, food, and food-environmental isolates from the Food and Drug Administration Center for Food Safety and Applied Nutrition (FDA-CFSAN) in-house collections and the Sequence Read Archive (SRA) database of NCBI, which are mainly isolates sequenced by the Center for Disease Control and Prevention (CDC) [43] (Additional file 1). Clinical isolates (total *n* = 79, including reference isolates described below) were selected to represent the phylogenetic diversity present in *L. monocytogenes* including isolates from lineages I, II, and III. A phylogenetic tree containing all *L. monocytogenes* isolates (*n* = 2012) from the GenomeTrakr Project (Genbank Bioproject PRJNA215355, November, 2014) was constructed. Clinical isolates, defined as such by two attributes in the Genbank Biosample Database, attribute package = clinical/host-associated and isolation source = human, were selected from each major clade (data not shown). Metadata for all isolates including isolation source and date are presented in Additional file 1. Food isolates (*n* = 95) include those from fresh produce, meat products (pork, poultry, beef), and dairy products (butter, milk, cheese), and industrial isolates consist of swabs of non-food contact surfaces from various meat production facilities. Food and environmental isolates were obtained from several randomly sampled United States Department of Agriculture (USDA) collection efforts, and no samples were epidemiologically linked to any cases of foodborne illness (P. Evans, USDA, personal communication). This set of food and meat-production facility isolates will be referred to broadly as food isolates in the remainder of the paper. Food isolates were also examined to ensure they represent the phylogenetic diversity of *L. monocytogenes*.

As outgroups for the phylogeny in Additional files 3 and 4, we included two previously sequenced *L. innocua* strains: ATCC33091 and FSL J1–023 [44]. *L. monocytogenes* reference genomes included EGD-e [45], EGD [46], and the high quality draft genomes SLCC2372, SLCC2540 [13], and FSL R2–503 [44]. Reference genome sequences were downloaded from Genbank [47]. A total of 176 isolates are described in Additional file 1, including two *L. innocua* isolates used only in the phylogenetic analysis. The remaining 174 isolates are included in the comparative analyses.

### Isolate culture and DNA extraction

Each strain was plated onto Trypticase Soy Agar and incubated overnight at 37 °C. Cells were then inoculated into Trypticase Soy Broth for DNA extraction using the DNeasy Blood and Tissue Kit (Qiagen, Valencia, CA, USA).

### Serotyping

In silico serotyping was performed first by BLAST analysis of presence/absence of previously identified genomic serotype markers [48]. When atypical presence/absence patterns were observed, in silico multilocus sequence typing (http://bigsdb.pasteur.fr/listeria/listeria.html) was used to help determine the serogroup.

### Determination of clonal complex

ST and CC for these genomes were determined using the sequence query tool built in the Pasteur Listeria MLST database (http://bigsdb.pasteur.fr/listeria/listeria.html) on the basis of the definition by Ragon et al. [11].

### Sequencing and assembly

Libraries were constructed for all FDA isolates listed in Table 1 using the Nextera XT Sample Preparation Kit (Illumina, Inc., San Diego, CA, USA), except for CFSAN028538 and CFSAN028542, which were previously constructed with the TruSeq DNA Library Preparation Kit (Illumina, Inc., San Diego, CA, USA). Paired-end DNA sequencing was performed on the Illumina MiSeq (Illumina, Inc., San Diego, CA, USA). Coverage was at least 50× for all isolates. All sequences were assembled using SPADES v.3.0.0 and contigs composed of less than 500 bp were excluded from the final assemblies. ORFs from each genome were annotated using the Prokka pipeline [49].

**Table 1:** Percent of clinical and food isolates containing significant genes in lineages I and II, and percent of outbreak isolates in lineages I and II containing significant genes

Percent of isolates containing gene								
Gene Name (PROKKA)	lin I clinical (*n* = 41)	lin I food (*n* = 34)	lin II clinical (*n* = 35)	lin II food (*n* = 60)	lin I clincal+ food (*n* = 75)	lin II clinical+food (*n* = 95)	lin I outbreak isolates (*n* = 27)	lin II outbreak isolates (*n* = 22)
cadC	30.95%	45.45%	20.00%	73.33%*	37.33%	53.68%	48.15%	13.64%
cadA	30.95%	45.45%	20.00%	73.33%*	37.33%	53.68%	48.15%	13.64%
hypothetical protein 1	21.43%	33.33%	14.29%	66.67%*	26.67%	47.37%	18.52%	0.00%
hypothetical protein 2	23.81%	33.33%	14.29%	66.67%*	28.00%	47.37%	18.52%	22.73%
hypothetical protein 3	21.43%	30.30%	8.57%	63.33%*	25.33%	43.16%	22.22%	13.64%
ebrB	2.38%	21.21%	8.57%	53.33%*	10.67%	36.84%	44.44%	4.55%
qac	2.38%	21.21%	5.71%	50.00%*	10.67%	33.68%	44.44%	4.55%
regulatory gene	2.38%	21.21%	8.57%	51.67%*	10.67%	35.79%	44.44%	4.55%

Asterisks represent statistically significant results (statistics not performed in outbreak isolates, thus, these results are reported only as percentages)

CFSAN022990 and CFSAN004330 were also sequenced on the PacBioRS II (Pacific Biosciences; Menlo Park, CA, USA) using standard protocols in order to identify the location of genes with high confidence. De-novo assembly was performed using the Hierarchical Genome Assembly Process (HGAP) with default parameters [50].

### Phylogeny reconstruction

A maximum likelihood phylogeny of *L. monocytogenes* based on a 95% majority SNP matrix was reconstructed using kSNP v2.1.2, with kmer size =19, as identified by the Kchooser script [51], and *L. innocua* as the outgroup.

### Comparative genomics

Orthologous coding sequence clusters were identified bioinformatically with OrthoMCL [52] using default parameters and a Markov inflation parameter of 1.5 [44]. Coding sequences present in only a single genome, singletons, were identified using the orthomclSingletons perl script included in the OrthoMCL package. Singletons were excluded if they are less than 50 amino acids in length, and were added into existing clusters if they have a sequence similarity of 60% or higher, coverage greater than or equal to 80%, and an e-value less than or equal to 10^− 5^ when searched against sequences in each cluster using a protein BLAST. Orthologous clusters were used to determine the core and accessory genomes of *L. monocytogenes.* The core genome is defined as the set of genes present in all of the genomes analyzed, while the accessory genome is defined as the set of genes that were missing from at least one genome. A presence absence gene matrix was constructed from the OrthoMCL output.

### Occurrence of virulence and stress response genes across all genomes

To determine the presence or absence of a previously described set of 125 virulence genes [22] we performed a nucleotide BLAST of each gene against each genome assembly. Significant hits were defined as those with coverage of at least 80% and a percent identity greater than or equal to 80%. All matches under this criteria also had an e-value less than or equal to 1e-100.

We also defined a set of 60 genes important in stress responses to different environmental factors (heat, cold, oxidative, acid, and general) [33, 34, 53–61] (Additional file 2), and performed a nucleotide BLAST search of these genes against each genome assembly as above.

To determine the presence of truncations in virulence and adaptation genes, we translated sequences to amino acids, aligned with MUSCLE [62], and manually inspected for truncations. Truncations were defined as present if a sequence was missing at least ten amino acids from the end of the sequence as compared to the EGD-e reference sequence. Nucleotide sequence location within each contiguous sequence (contig) of the assembly was examined to ensure that the truncation did not occur due to its position at the ends of contigs.

### Determination of enriched genes in clinical, food, and environmental isolates

We performed a Yates-corrected chi-square test with a Bonferroni correction for multiple testing on each orthologous gene cluster to determine whether members of a cluster are significantly associated (*p* < .05) with either clinical or food isolates (*n* = 41 lineage I clinical isolates, *n* = 34 lineage I food isolates, *n* = 35 lineage II clinical isolates, *n* = 60 lineage II food isolates). We chose the conservative Bonferroni correction in order to reduce the probability of Type I errors in our analyses. We removed core genes from this analysis as well as genes that were present in less than 15% of all isolates.

### Determination of enriched SNPs in clinical, food, and environmental isolates

Using the SNP matrix produced by kSNP, we used the Predict Phenotype from SNPs (PPFS2) (https://sourceforge.net/projects/ppfs/files/) add on package to kSNP [63] to determine whether any SNPs could be used to correctly predict a clinical or food phenotype. PPFS2 identifies SNPs that are not randomly assigned to a phenotype based on the chi square probability. In brief, diagnostic SNPs were identified using the PPFS2 programs PickPhenotypeSubset, GetSNPprobs, and DiagnosticSNPs. GetSNPprobs is used to calculate the chi square probability that SNP alleles are distributed randomly with respect to phenotype for each SNP identified by kSNP. DiagnosticSNPs sorts the list of SNP probability values output by GetSNPprobs. Starting with the SNP with the lowest *p*-value, DiagnosticSNPs calculates the accuracy, positive predictive value (PPV), and negative predicted value (NPV) (see full reference [57] for details). SNPs are then sequentially added from the sorted list, and accuracy, PPV, and NPV are recalculated. This is repeated until the accuracy, PPV, and NPV values decline, at which point the SNPs used in the calculation are defined as diagnostic SNPs. We determined the diagnostic SNPs for lineage 1, lineage 2, and across all *L. monocytogenes* lineages.

## Results

### Identification of virulence and stress tolerance gene clusters

The core genome of 174 isolates consists of 2322 genes, the accessory genome 2381 genes, and the pan genome 4703 genes, which is in the order of prior core genome calculations for *L. monocytogenes* [22, 44]. Also similar to previous studies, we identified variations among the virulence genes of our dataset, although none are significantly associated with either clinical or food isolates [14, 22, 38] (Additional file 3). Of the 125 virulence genes examined, 45 genes are either missing or truncated in some isolates (Additional file 3). The presence of pathogenicity island 1 (LIPI-1) is highly conserved among all *L. monocytogenes* isolates, with the exception of hemolysin LLO, which is truncated in two genomes. A five-gene stress survival islet (SSI_1) is missing in several lineage I and II isolates as previously observed [14, 22, 38]. The presence of pathogenicity island LIPI-3 (LIPI-3) is variable among lineage I isolates, and surprisingly, was also detected in a single isolate from lineage II. Although it has been detected in *L. innocua* [64], a complete LIPI-3 has not been previously detected in lineage II *L. monocytogenes* isolates, although a partial LIPI-3 is present in several lineage II isolates [38]. Some virulence loci occur more frequently among lineage I or lineage II isolates, suggesting that they may be more important for virulence in one or the other lineage. Lmo1082, previously found to be up-regulated in vivo during infection in EGD-e [65], occurs more frequently among lineage II isolates than lineage I isolates (88% and 57%, respectively). In contrast, lmo0320, an LPXTG surface protein required for entry into some mammalian cells [66], is present in higher frequency among lineage I food isolates than lineage II food isolates (100% and 56%, respectively). Other genes are missing from taxa in both lineages, including lmo0263 (*inlH*), lmo0478, and lmo2558, suggesting a less essential role in virulence for these loci than for those loci which are conserved across all taxa. Internalin A is truncated more frequently in food isolates than in clinical isolates as has been reported previously; truncations occurred in 36% of all food isolates averaged across lineages I and II [67]. No other truncations in virulence genes were significantly associated with isolation source.

Stress response genes are highly conserved among all lineages and both isolation sources, even more so than the virulence genes (Additional file 4). A single significant stress tolerance gene, *cadA*, important for cadmium resistance, is significantly associated with food isolates in lineage II. No truncations are associated with a single isolation source, but truncations in lmo1589, *argB*, and lmo1433 are frequently present in lineage I isolates, but not lineage II.

### Analysis of gene clusters for significant association with isolation source

We analyzed OrthoMCL gene clusters to search for additional genes that may be significantly associated with isolation source. Clusters containing core genes or genes present in less than 15% of all isolates were not considered. In lineage I no clusters were identified, but eight clusters are significantly associated with food isolates in lineage II out of 778 gene clusters analyzed (Table 1, Additional file 5, Additional file 6). The eight significant lineage II clusters contain genes encoding for three hypothetical proteins (referred to here as hypothetical protein genes 1–3,), as well as the cadmium resistance genes *cadA* and *cadC* (*cadA* noted above), the multi-drug resistance gene *ebrB*, the *qac* gene involved in resistance to quaternary ammonium compounds, and the nucleoid occlusion factor *slmA* (sequences listed in Additional file 7). Seven of the eight significant gene clusters identified by OrthoMCL contain multiple homologous sequences; *cadA* and *cadC* clusters each consist of seven homologues, gene clusters encoding hypothetical proteins 1 and 2 each contain three homologues, the gene cluster encoding *ebrB* and *qac* has four and three homologues, respectively, and the *slmA* gene cluster has two.

To assess the accuracy of the PROKKA annotations for the genes identified in lineage II clusters, we compared the sequences and their homologues to published gene sequences. Four cadmium resistance cassettes have been described in *L. monocytogenes*, each of which consists of a P-type ATPase, *cadA*, and its putative transcriptional regulator, *cadC*. Genes from the same cassette occur as a pair within a genome, e.g., *cadA1* occurs with *cadC1, cadA2* occurs with *cadC2*, etc.; and *cadAC* is used to designate the pair. In our data, the four *cadA* and *cadC* homologues share 100% nucleotide sequence similarity with *cadA1-cadA4* from *L. monocytogenes*. The three additional homologues, referred to here as *cadA* and *cadC* (5–7), contain the same NCBI CDD domains as those found in *cadA1-A4*. The multidrug resistance gene *ebrB* has not been characterized in *Listeria*, but the *ebrB* homologues identified here contain the same domain as *ebrB* from *Bacillus* (Name: emrE; NCBI CDD Accession: COG:2076)*.* All homologues in the cluster annotated as *qac* also contain this domain, as do published *qacC* sequences from *Staphylococcus aureus.* Sequences of homologues from the *ebrB* and *qac* clusters show 41–52% sequence identity at the amino acid level when compared against each other. In contrast, the sequences in the cluster annotated as *slmA* do not contain the same domains as *slmA* characterized from *E. coli*. Sequences in this study designated as *slmA* and *slmA* from *E. coli* both share the TetR_N superfamily domain, but our homologues contain the domain acrR (NCBI CDD Accession: COG1309) instead of the domain *slmA* (NCBI CDD Accession: PRK09480). The correct annotation for these sequences is unclear, but we assume that they have a regulatory role based on the presence of the acrR domain (DNA-binding transcriptional regulator, AcrR family). This cluster will therefore be referred to as the “regulatory gene cluster” in the remainder of the discussion.

The function of the three genes encoding hypothetical proteins is unknown. Using BLAST, we searched each sequence against the non-redundant (nr) database, but hits consist only of other genes encoding hypothetical proteins in *L. monocytogenes* plasmids. These results suggest that the genes may be plasmid-borne. There are no hits when searching against the COG and PFAM databases.

All eight gene clusters found significantly associated with lineage II food isolates are likely plasmid encoded, although some *cadAC* homologues may be found on other mobile elements. We performed a BLAST search of the most abundant homologue of each of the eight genes against the nr database, and found that the top hits for each sequence are closed plasmids (coverage of 99–100%, percent identity 99–100%). Furthermore, we performed a nucleotide BLAST of the genes against 27 publicly available closed *Listeria* plasmids from Genbank (October 2017) (Additional file 8). All but a single plasmid contain at least one homologue of *cadAC*, and 25/27 contain genes encoding hypothetical proteins 2 and 3 (sequence similarity greater than 60% and coverage of at least 80%). The gene encoding hypothetical protein 1 is present in 15/27 plasmids, while *ebrB, qac*, and the regulatory gene are present in 8/27. The conservation of the genes across diverse plasmids suggest they may confer a selective advantage. To verify the position of the eight genes in isolates of our dataset, we closed the genomes of CFSAN0022990 and CFSAN004330, two food isolates from this study, using PacBio technology. CFSAN022990 bears a single plasmid containing all eight significant genes (99% similar to reference genome *L. monocytogenes* 6179, 90% coverage), as does CFSAN004330, albeit different homologues.

### Presence of eight genes among serotypes

**Fig. 1:**
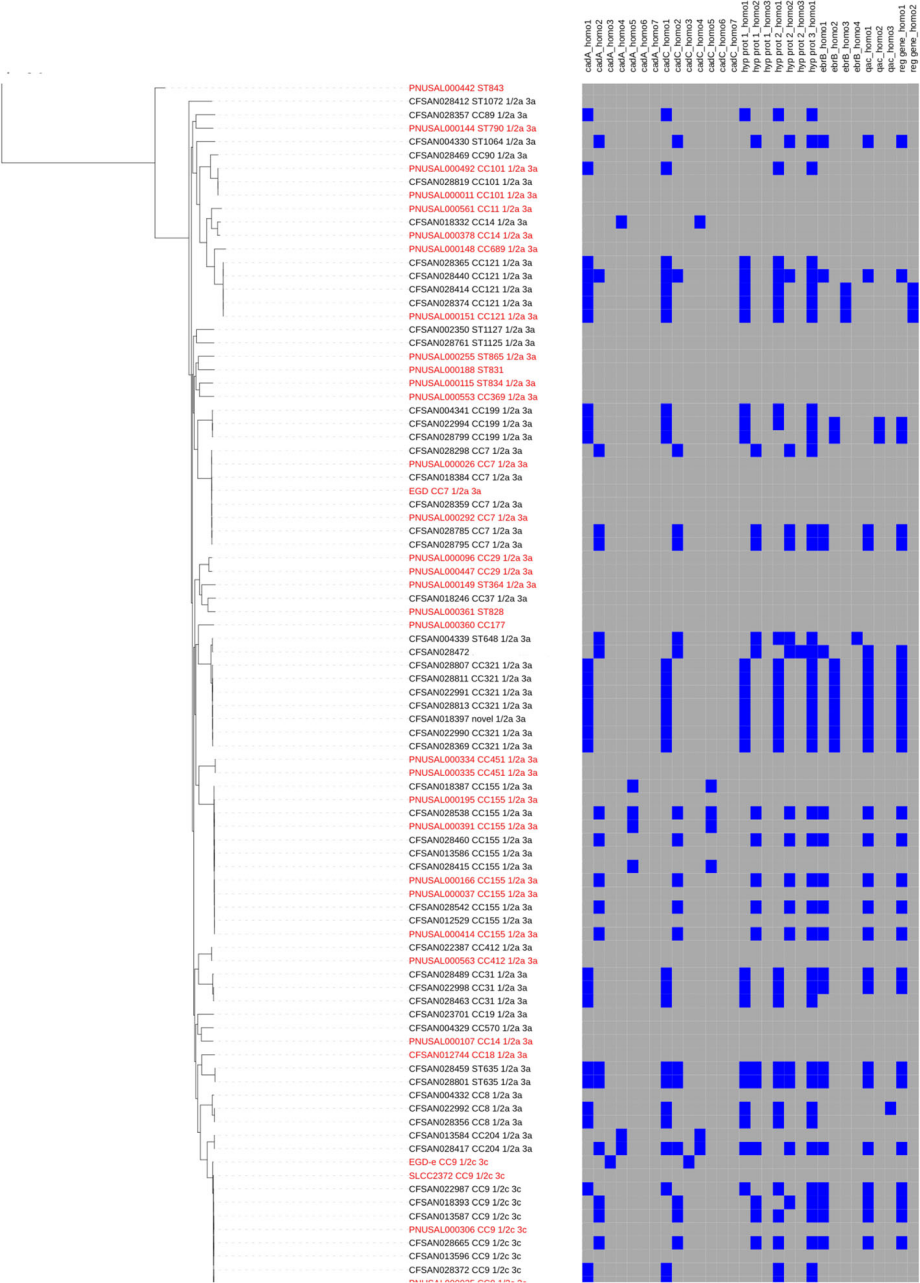
Phylogenetic distribution of eight significant genes in lineage II. Tree constructed by kSNP, 95% majority maximum likelihood (ML). Clinical isolates = red, food isolates = black, reference isolates not classified by isolation source = blue. Grey = gene absence, blue = gene presence

In lineage II, over half of serotype groups 1/2a plus 3a (referred to here as serotype group 1/2a) and 1/2c plus 3c isolates (serotype group 1/2c) contain at least two of the eight genes (41/77 and 10/15, respectively). In both serotypes, *cadAC1* and *cadAC2* predominate over other *cadAC* homologues, along with the primary homologues of genes encoding hypothetical proteins 1–3 when *cadAC1* is present and the second homologue of these proteins when *cadAC2* is present (Fig. 1, Additional file 6). In lineage I, the eight genes are present in (24/74) isolates able to be serotyped, and occur more frequently among serotypes 1/2b, 3b, and 7 (serotype group 1/2b), than among 4b, 4d, 4e (serotype group 4b) isolates (18/35 and 6/34, respectively), and none of the five 4b variant (4bv) isolates contained any of the genes. The proportion of homologues of each of the eight genes present in 1/2b isolates is similar to that of lineage II food isolates. In the few 4b isolates containing any of the genes, the homologues of *cadAC* vary (2 *cadAC1*, 1 *cadAC2*, 3 *cadAC4*, and 1 *cadAC6*), and most isolates do not have homologues of any of the three hypothetical protein encoding genes, the *qac* genes, or the regulatory gene (Additional file 6). Please note, references to serotype groups include clinical and food isolates.

### Estimation of identified genes in outbreak isolates

We determined how frequently the eight significant genes from lineage II occurred in a set of outbreak isolates from lineages I (*n* = 27) and II (*n* = 22) compiled from a recent publication and our internal database [27] (Additional file 9). Consistent with the low frequency of the eight genes in lineage II clinical isolates, only 4/22 lineage II outbreak isolates contain any of the eight genes, and none contain the gene encoding hypothetical protein 1 (Table 1).

### Identification of SNPs

No SNPs in any core genome gene are statistically significantly nonrandomly associated with food or clinical isolates in lineages I or II. Several SNPs are statistically significantly associated with food or clinical isolates in the accessory genome, but both SNP alleles of all SNPs are associated with a single isolation source, while the SNP is often absent from the genomes of the other isolation source. Thus, there are no informative alternative SNP nucleotide states. Furthermore, all SNPs occur either within or between genes identified in the gene cluster analyses, or just below the threshold of significance. Thus, we do not feel that these SNPs provide much novel information not already discussed elsewhere in the paper and thus they are not reported here.

## Discussion

We identify eight genes that are significantly associated with food isolates across lineage II. This is the first comparative genomics study to identify genes that are significantly associated with diverse food isolates in *L. monocytogenes*, although one benzalkonium resistance gene was found to be associated with food-associated *L. monocytogenes* sublineages [38]. In lineage I, the eight genes are present in an overall lower frequency than in lineage II, and are not significantly associated with either food or clinical isolates. The distribution of homologues of the eight genes varies with serotype in lineage I. Homologues present in serotype group 1/2b were similar to those of lineage II food isolates, while serotype group 4b *cadAC* homologues were varied and isolates were often missing the three hypothetical protein encoding genes found nonrandomly associated with lineage II food isolates. Previous studies showed that *cadAC1* occurs less frequently in 4b isolates than in 1/2b isolates [68], and *cadAC4* is present in a higher proportion of 4b isolates than other serotypes [69].

Given the high degree to which the presence of these eight genes is conserved among phylogenetically diverse food isolates in lineage II, it is likely that the genes play an important role in the survival and proliferation of *L. monocytogenes* in the food environment. Understanding the mechanisms that underlie these processes is critical for the development of management practices to reduce *L. monocytogenes* in the food supply. In addition, functional information may be useful in predicting the risk associated with certain foods. This is particularly important as recent outbreaks have occurred in foods previously categorized as low risk such as ice cream and produce [42].

Collectively, little is known about the function of the eight genes. The heavy metal transporting efflux pump *cadA* and its putative repressor *cadC* expel toxic cadmium from the cell [70], while multidrug resistance genes such as *ebrB* and genes encoding for resistance to quaternary ammonium compounds commonly used as industrial cleaners also reduce the level of toxic compounds in the cell [71]. Thus, the presence of *cadAC*, *ebrB,* and *qac* in lineage II food isolates likely promotes survival in food and in the food-processing environment by mitigating cell exposure to harmful chemicals. No information is available regarding the function of the three genes encoding hypothetical proteins or the acrR domain containing regulatory gene.

It is less clear why the eight loci are found in low frequencies among lineage II clinical isolates. Antibiotic resistance is often associated with infection [72]. Some of the top-infecting *L. monocytogenes* serotypes 1/2a, 1/2b, and 4b [68] and outbreak isolates, as well as 1/2b isolates in this study, contain *cadA1* and *cadA2*. Furthermore, as a facultative pathogen, *L. monocytogenes* spends part of its life cycle in the environment before infecting a host, thus clinical isolates should also benefit from the genes. It is possible that the genes and/or mobile elements on which they reside confer a selective disadvantage within the host that outweighs any advantage gained while in the environment, or perhaps clinical isolates are transient in the environment, with few opportunities to acquire foreign DNA from other environmental isolates. The functions of the genes in lineages I and II may also vary against the different genetic background of the two lineages.

The function of *cadAC* beyond cadmium resistance has not been well explored, and studies do suggest that some *cadAC* homologues have a role in virulence. The expression of the negative regulator *cadC3* in EGD-e (lmo1102) is up regulated during in vivo infection and was determined to be essential for virulence [65]. However, a recent study showed that the inactivation of *cadA4* enhances virulence in a *Galleria* insect model [69], and in vivo, CadC3 is required for *L. monocytogenes* infection [73].

Given the presence of the eight genes on mobile elements, particularly plasmids, we were interested in testing whether plasmids are more frequent among food isolates than among clinical isolates in lineage II. To do this, we performed a nucleotide BLAST search of 27 closed *L. monocytogenes* plasmids against our genomes. Genomes of only 6/36 clinical isolates have sequences similar to the plasmids, compared to 38/60 food isolates (sequence similarity greater than 60% and plasmid coverage of at least 20%). Correlations between plasmid presence and serotype have been noted previously in *L. monocytogenes*. In a group of 173 isolates collected in France, food and environmental isolates from serogroups 1 and 4 harbored plasmids more frequently than clinical isolates [74, 75]. In a collection of 322 UK isolates, plasmids were more frequently identified in serotype 1/2a food isolates than in clinical isolates, whereas the converse was true for serotype 1/2c [75]. Mobile genetic elements (MGE) such as plasmids expand the coding potential of the accessory genome, and increase bacterial fitness under some conditions. Thus, plasmids in food isolates likely provide a source of novel genetic information to adapt to diverse conditions encountered in food environments.

Although no statistically significant SNPs are reported here, additional studies which examine polymorphisms in groups of clinical and food isolates may yield insights into the mechanisms of adaptation or genetic markers to differentiate the two groups. For example, comparing the occurrence of SNPs among each core and accessory gene may identify genes with a higher frequency of mutation in either clinical or food isolates which could reflect the effect of different selection pressures on the two groups [76]. Assessing polymorphism differences between multiple pairs of genetically similar clinical and food isolates may identify markers to distinguish these isolates, as was demonstrated in a recent study which identified several SNPs which could differentiate adherent-invasive *E. coli* (AIEC) from non-AIEC strains [77]. In addition, other nonrandomly distributed polymorphisms such as insertions and deletions (indels) may be present in core genes and should be assessed in future studies utilizing reference-based methods.

## Conclusions

Eight genes are significantly associated with food isolates in lineage II. Intriguingly, the eight genes are virtually absent in outbreak isolates from lineage II, are composed of homologues which show a unique distribution among serotypes in lineage I, and are highly conserved across 27 closed *L. monocytogenes* plasmids. Plasmids are also more frequently associated with lineage II food isolates than with clinical isolates. The role of the eight genes and plasmids in the survival and proliferation of *L. monocytogenes* in food and in the food-processing environment should be explored. This information is critical if we wish to control populations of *L. monocytogenes* in the food supply, particularly in light of the fact that recent *L. monocytogenes* outbreaks have occurred in foods that have historically been considered to be low risk. Future studies should assess these observations on a larger scale and explore the utility of the eight genes and their homologs in risk assessment models.

## Additional files

Additional file 1: List of 176 isolates included in this study and the associated metadata. (XLSX 23 kb)

Additional file 2: List of stress tolerance genes included in this study. (XLSX 33 kb)

Additional file 3: Phylogenetic distribution of 45/125 virulence genes across *L. monocytogenes* (genes which are conserved are not shown). Blue = present, yellow = absent, gray = truncated. Clinical isolates = red, food isolates = black, reference isolates not classified by isolation source = blue. (TIF 1165 kb)

Additional file 4: Phylogenetic distribution of 7/65 stress tolerance genes which vary across *L. monocytogenes,* (conserved genes not shown). Green = present, purple = absent, dark purple = truncated. Clinical isolates = red, food isolates = black, reference isolates not classified by isolation source = blue. (TIF 1104 kb)

Additional file 5: Chi-Square test for significant gene clusters in lineage II. (XLSX 49 kb)

Additional file 6: Presence-absence matrix of eight genes across isolates in this study. Serotype, CC or ST, and the presence of a plasmid is indicated for each genome. (XLSX 37 kb)

Additional file 7: List of eight significant genes, their homologues, and sequence accessions. (XLSX 18 kb)

Additional file 8: List of plasmids downloaded from Genbank. (XLSX 9 kb)

Additional file 9: List of outbreak isolates. (XLSX 11 kb)

## Abbreviations

CDC: Center for Disease Control and Prevention
contig: Contiguous sequence
FDA-CFSAN: Food and Drug Administration Center for Food Safety and Applied Nutrition
HGAP: Hierarchical Genome Assembly Process
LIPI-3: Pathogenicity island LIPI-3
ML: Maximum likelihood
NCBI: National Center for Biotechnology Information
NPV: Negative predicted value
PPFS2: Predict Phenotype from SNPs
PPV: Positive predictive value
SNP: Single nucleotide polymorphism
SRA: Sequence Read Archive
tRNA: Transfer RNA
USDA: United States Department of Agriculture
WGS: Whole Genome Sequencing
