# Supplementary figures and images for: Genes significantly associated with lineage II food isolates of Listeria monocytogenes
Source: BMC Genomics. 2018 Sep 25;19:708. doi: 10.1186/s12864-018-5074-2 (PMC6157050; doi:10.1186/s12864-018-5074-2)

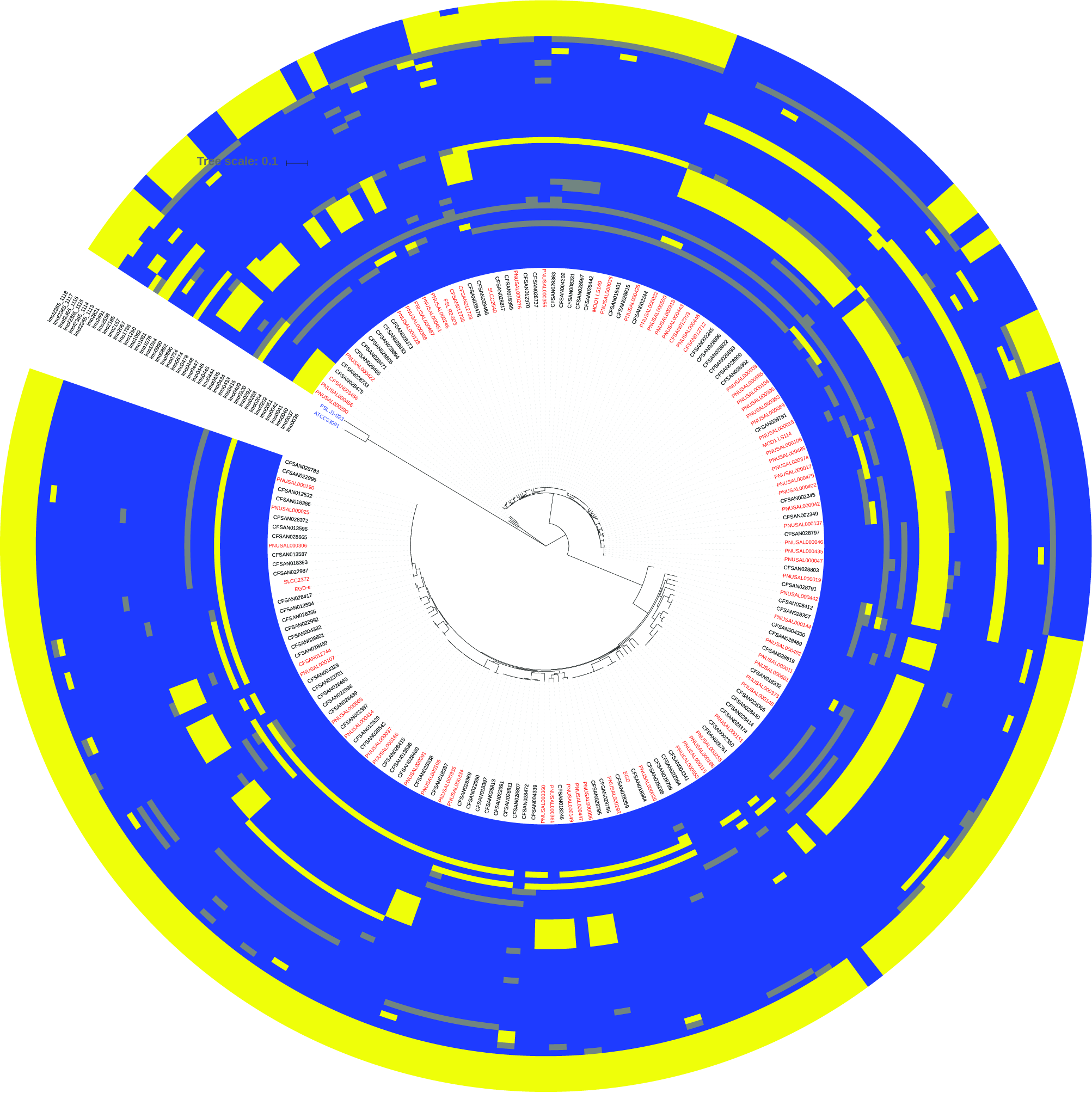

Supplement: Supplementary file 3 — Phylogenetic distribution of 45/125 virulence genes across L. monocytogenes (genes which are conserved are not shown). Blue = present, yellow = absent, gray = truncated. Clinical isolates = red, food isolates = black, reference isolates not classified by isolation source = blue. (TIF 1165 kb) [file 12864_2018_5074_MOESM3_ESM.tif]

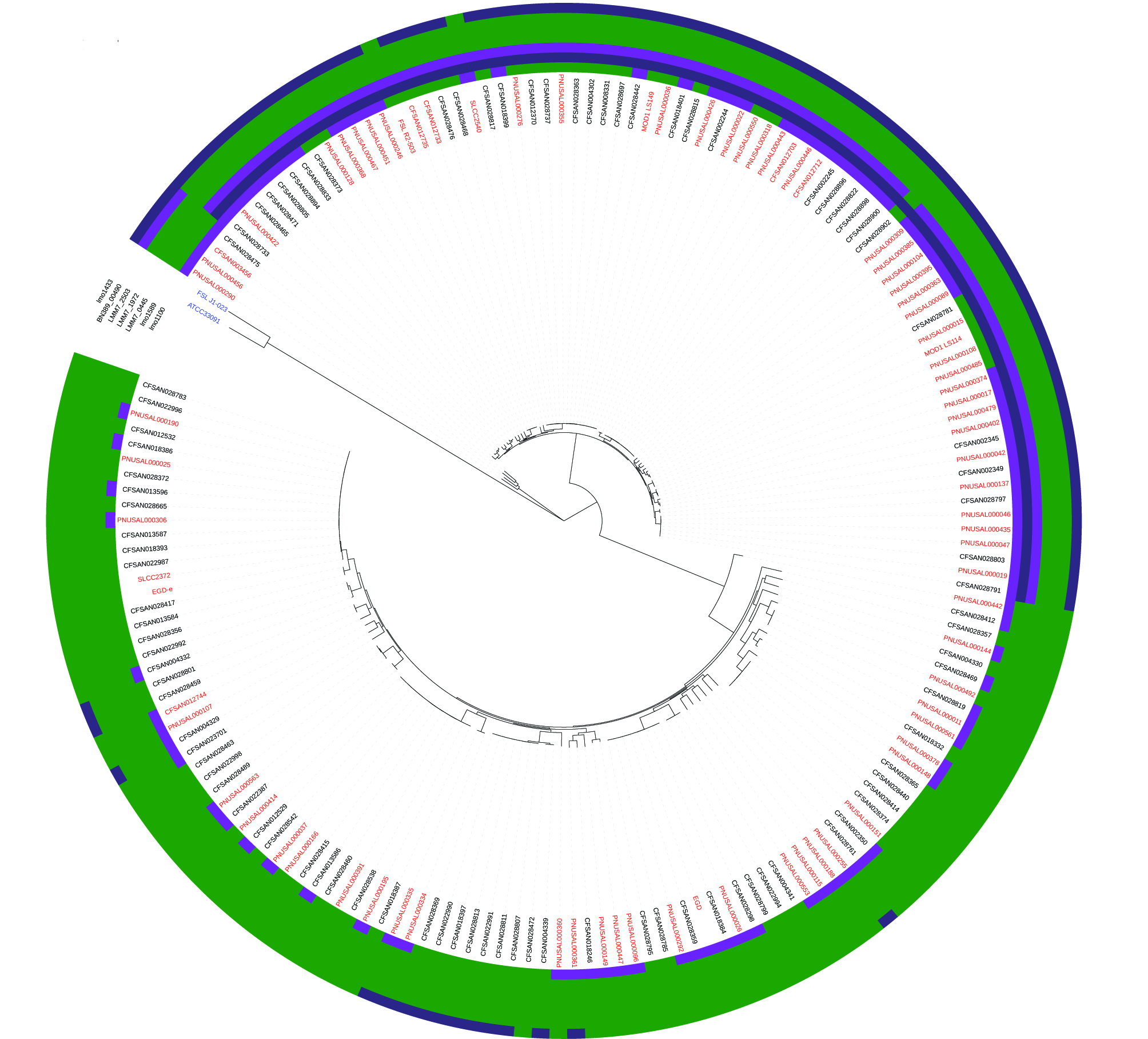

Supplement: Supplementary file 4 — Phylogenetic distribution of 7/65 stress tolerance genes which vary across L. monocytogenes, (conserved genes not shown). Green = present, purple = absent, dark purple = truncated. Clinical isolates = red, food isolates = black, reference isolates not classified by isolation source = blue. (TIF 1104 kb) [file 12864_2018_5074_MOESM4_ESM.tif]
